# Case Report: Locally advanced lung adenocarcinoma with a novel MIR217HG-ALK rearrangement responding to neoadjuvant alectinib

**DOI:** 10.3389/fphar.2025.1602654

**Published:** 2025-08-11

**Authors:** Yinyin Xue, Wen Li, Kaili Huang, Qinghua Zhou, Qiang Wu

**Affiliations:** ^1^ Department of Radiation Oncology Cancer Center, West China Hospital, Sichuan University, Chengdu, Sichuan, China; ^2^ Lung Cancer Center/Lung Cancer Institute, West China Hospital, Sichuan University, Chengdu, Sichuan, China; ^3^ Department of Medical Oncology Cancer Center, West China Hospital, Sichuan University, Chengdu, Sichuan, China; ^4^ Department of Thoracic Surgery, West China Hospital, Sichuan University, Chengdu, Sichuan, China

**Keywords:** lung adenocarcinoma, MIR217HG-ALK, neoadjuvant alectinib, major pathological response (MPR), stage IIIB-N3

## Abstract

**Background:**

Anaplastic lymphoma kinase (ALK) rearrangement is a crucial oncogenic driver in non-small cell lung cancer (NSCLC). ALK tyrosine kinase inhibitors (ALK-TKIs) represent the primary therapeutic option for advanced NSCLC patients with ALK rearrangements. However, the definitive determination of ALK-TKIs sensitivity towards newly identified rare ALK rearrangements remains elusive. Herein, we present a patient with lung adenocarcinoma harboring a novel ALK rearrangement who exhibited major pathological response (MPR) following neoadjuvant treatment with alectinib.

**Materials and methods:**

We conduct immunohistochemistry (IHC) staining with Ventana with D5F3 clone, fluorescence *in situ* hybridization (FISH, The Vysis ALK Break Apart FISH Probe Kit), and next-generation sequencing (NGS) analyses (Burning Rock, Guangzhou, China) on biopsy sample obtained from the patient.

**Results:**

The patient, a 66-year-old female, was diagnosed with stage IIIB-N3 adenocarcinoma in the right upper lobe of the lung. NGS testing revealed a previously unreported MIR217HG-ALK rearrangement, which was subsequently confirmed by IHC and FISH. Following 5 months of neoadjuvant treatment with alectinib, the patient underwent the right upper lobectomy and achieved MPR, resulting in disease-free survival (DFS) exceeding 19 months.

**Conclusion:**

In this study, we present the first documented case of a patient with lung adenocarcinoma harboring a novel MIR217HG-ALK rearrangement who exhibited favorable response to neoadjuvant alectinib. Our findings suggest that alectinib holds promise as an efficacious therapeutic option for individuals with MIR217HG-ALK rearranged lung adenocarcinoma, thereby offering valuable insights for the clinical management of these patients.

## Introduction

For advanced non-small cell lung cancer (NSCLC) with anaplastic lymphoma kinase (ALK) positivity, ALK-tyrosine kinase inhibitors (ALK-TKIs) are considered the preferred first-line treatment option (Mok et al., 2020). The Echinoderm microtubule-associated protein-like 4 gene (EML4)-ALK rearrangement is one of the predominant fusion partners in NSCLC, and currently, numerous novel ALK fusion partners have also been identified (Sasaki et al., 2010; Ou et al., 2020). However, considering that these newly discovered ALK fusion partners may exhibit distinct response profiles to ALK-TKIs (Chuang et al., 2021), it is imperative to identify additional rare ALK fusion genes to provide more valuable clinical evidence for patient management. Herein, we present a case of unresectable locally advanced lung adenocarcinoma harboring a novel MIR217HG-ALK rearrangement, and after receiving neoadjuvant treatment with alectinib, the patient achieved major pathological response (MPR) and demonstrated disease-free survival (DFS) exceeding 19 months without notable adverse reactions observed.

## Case report

**FIGURE 1:**
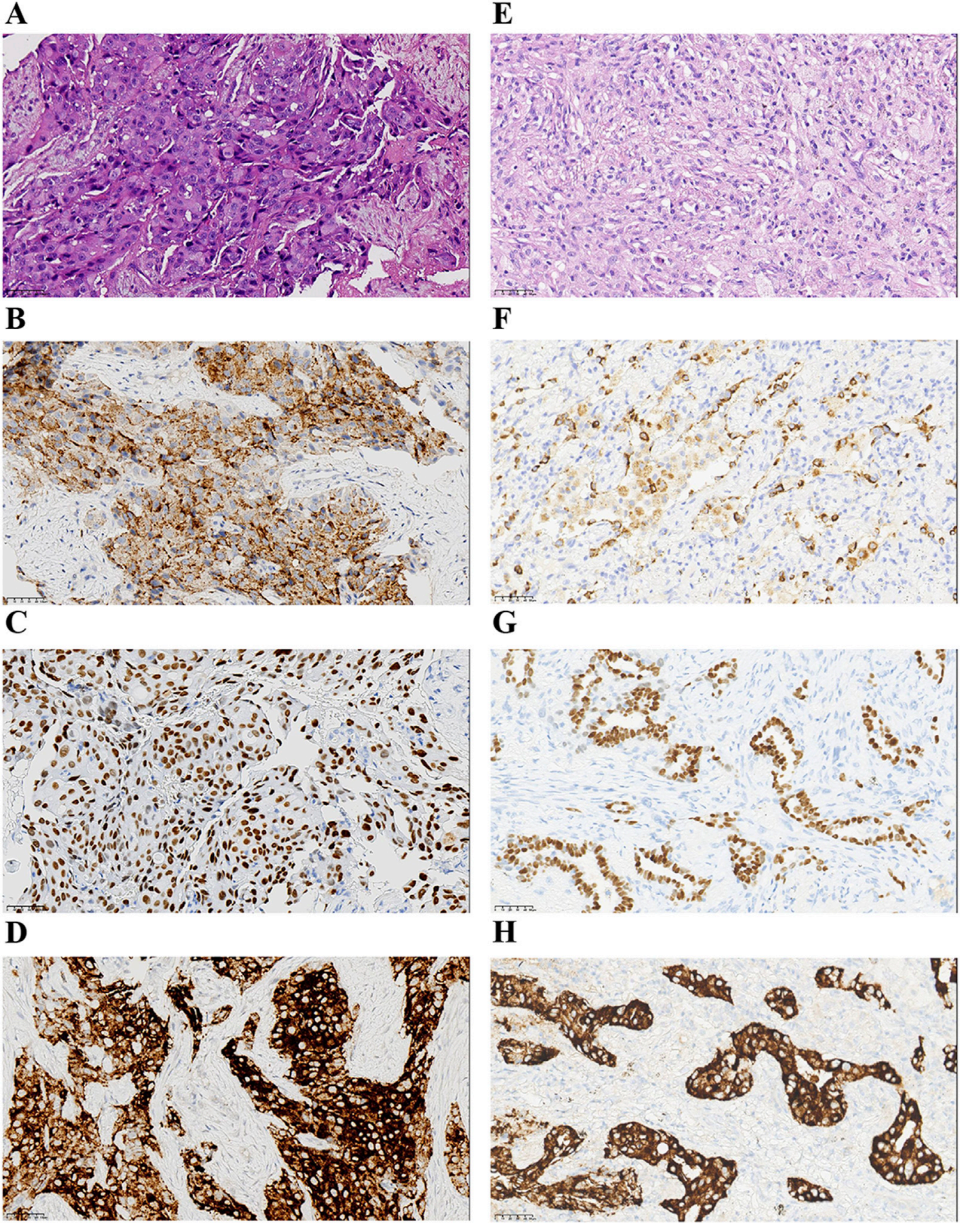
Pathological findings of the patient. **(A–D)** Hematoxylin-eosin staining and IHC staining at the time of diagnosis (x40). Hematoxylin-eosin staining of lung adenocarcinoma **(A)**. The IHC staining indicated positive for Napsin A **(B)**, TTF-1 **(C)**, and ALK protein expression **(D)**. **(E–H)** Findings of postoperative pathology examination (x40). Hematoxylin-eosin staining **(E)**, and the IHC staining of Napsin A **(F)**, TTF-1 **(G)**, and ALK protein expression **(H)**. IHC, immunohistochemistry; TTF-1, thyroid transcription factor-1; ALK, anaplastic lymphoma kinase.

**FIGURE 2:**
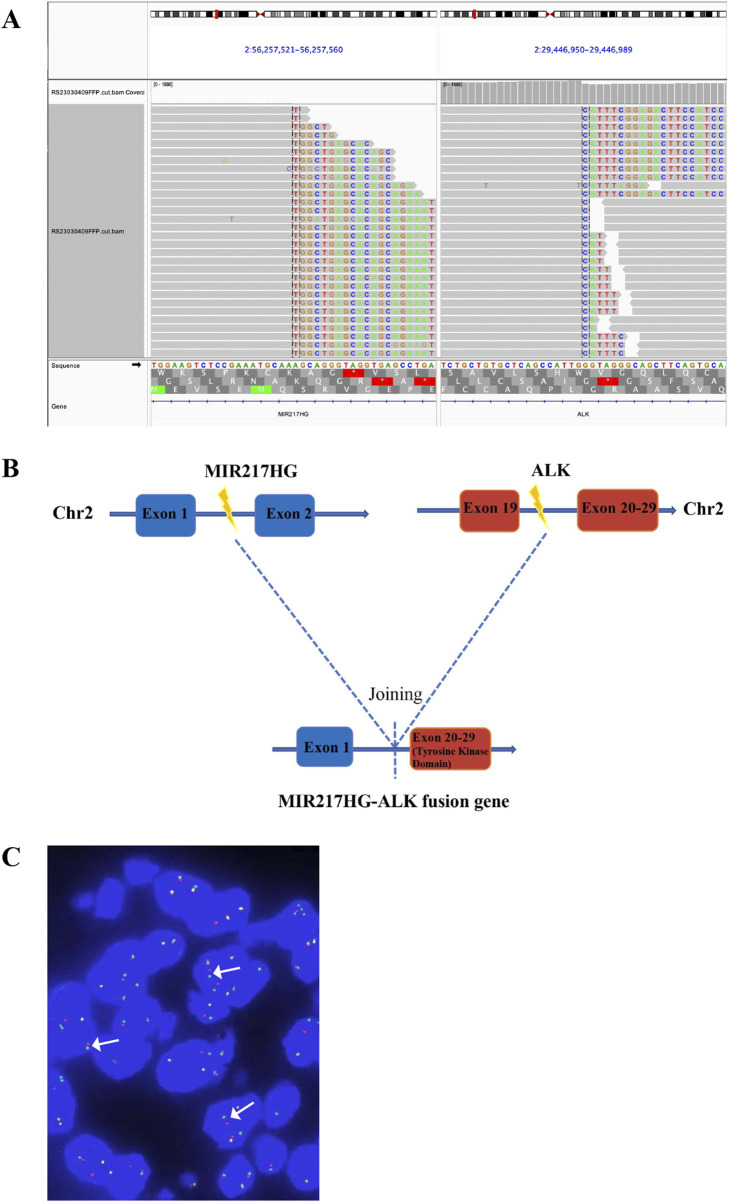
Identification of MIR217HG-ALK fusion by NGS. **(A)** The Integrative Genomics Viewer (IVG) snapshot of MIR217HG-ALK fusion. **(B)** Illustration of MIR217HG-ALK fusion (the new variant consists of MIR217HG exon 1 and ALK exon 20–29, positivity threshold: ≥15%). **(C)** FISH result. ALK gene isolation probe successfully identified the presence of ALK fusion (the percentage of the rearranged cells: 30.27%), displaying a split signal (either green or red, indicated by arrows) at both the 5′and 3′ends of the ALK gene. The yellow signals represent intact ALK gene. NGS, next-generation sequencing; ALK, anaplastic lymphoma kinase; FISH, fluorescence *in situ* hybridization.

**FIGURE 3:**
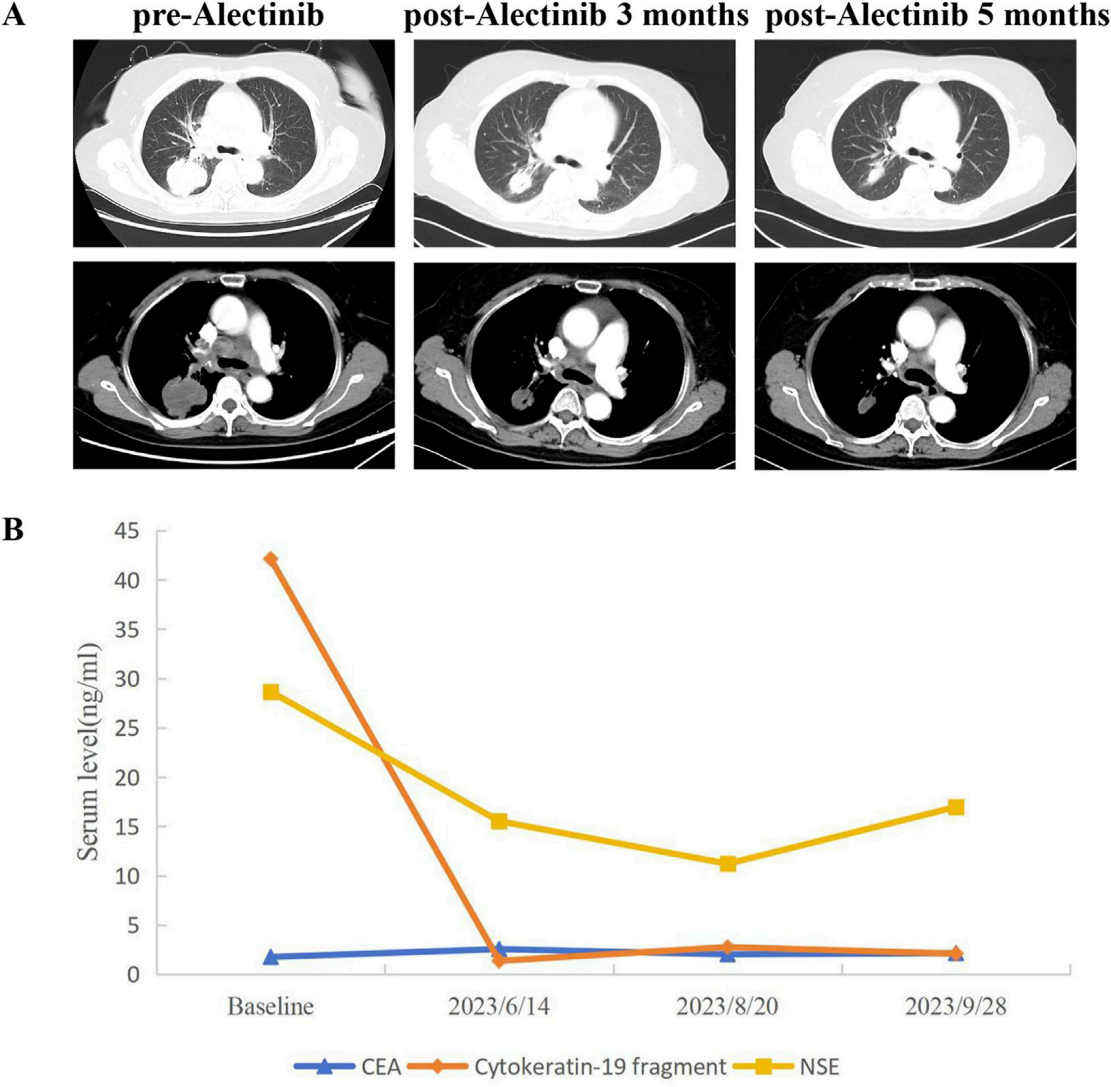
CT scans and changes of tumor markers during treatment. **(A)** Chest CT images of right lung adenocarcinoma before and after neoadjuvant alectinib (evaluated according to RECIST 1.1 criteria). **(B)** Dynamic monitoring of tumor markers during neoadjuvant alectinib. CT, Computed tomography.

**FIGURE 4:**
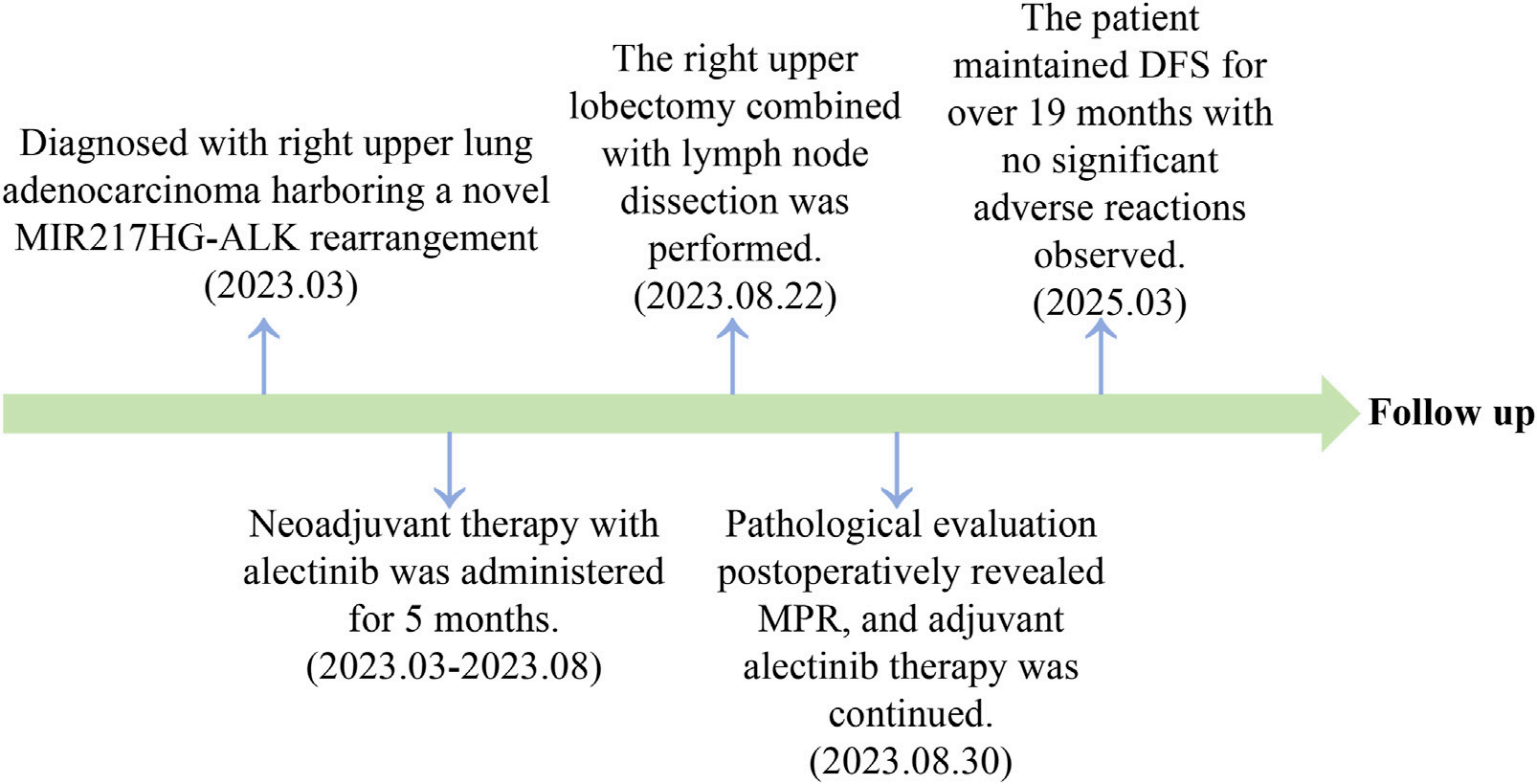
A timeline of major clinical events since diagnosis is provided.

A 66-year-old non-smoking female presented to our hospital with a 6-month history of persistent cough with sputum production. The patient denied any symptoms of chest tightness, chest pain, fever, or dyspnea. Her medical history was unremarkable, with no evidence of diabetes, hypertension, or cardiac disease, and no family history of malignancies. Chest contrast-enhanced computed tomography (CT) revealed a mass measuring approximately 5 cm × 4.9 cm in the posterior segment of the right upper lobe, accompanied by enlarged lymph nodes in the right hilum and bilateral mediastinum. Meanwhile, the patient underwent a thorough imaging evaluation including enhanced magnetic resonance imaging (MRI) of the head, single-photon emission computed tomography (SPECT) bone scan, and contrast-enhanced CT of the abdomen, which revealed no metastasis in the head, bones, liver, or adrenal glands. Bronchoscopic biopsy was performed and the pathological examination revealed invasive adenocarcinoma on the right upper lobe, and the patient was clinically classified as stage IIIB with involvement of contralateral mediastinal lymph nodes (cT2bN3M0, the eighth edition of the TNM classification for lung cancer). The immunohistochemistry (IHC) staining with Ventana with D5F3 clone demonstrated positive expression of thyroid transcription factor-1 (TTF1) and Napsin A (Figures 1A–C), indicating an original adenocarcinoma of the lung. Subsequently, next-generation sequencing (NGS) testing (Burning Rock, Guangzhou, China) was performed on the lung tissue obtained from bronchoscopy, revealing a novel MIR217HG-ALK fusion variant (M3′UTR: A20; abundance: 3.31%) with an intact ALK kinase domain and no TP53 mutation (Figures 2A,B). The MIR217HG gene, which serves as the host gene for MIR217, is a type of RNA gene classified as a long non-coding RNA (lncRNA). This novel ALK fusion variant was further confirmed through IHC and fluorescence *in situ* hybridization (FISH, The Vysis ALK Break Apart FISH Probe Kit) analysis (Figures 1D, 2C). Then, oral administration of alectinib dose of 300 mg twice daily was initiated for treatment from 14 March 2023. Three months later, chest contrast-enhanced CT demonstrated a significant reduction in the mass located in the right upper lobe, measuring approximately 3.9 cm × 2.2 cm, accompanied by shrinkage of the right hilum and bilateral mediastinal lymph nodes. Five months later, follow-up chest CT indicated continued reduction in the right upper lobe mass and the involved lymph nodes, with the mass size measuring approximately 3.1 cm × 2.0 cm (Figure 3A). Additionally, both CEA and cytokeratin-19 fragment levels showed a significant decrease (Figure 3B). Consequently, a surgical procedure involving the right upper lobectomy combined with lymph node dissection was performed on the patient on 22 August 2023. Postoperative pathological examination revealed that approximately 10% of the primary lesion consisted of residual tumor tissue, while the remaining portion comprised necrotic tissue, fibrosis, and inflammation (Figures 1E–H). A total of 13 lymph nodes were assessed for treatment response: cancer metastasis was observed in station 2 (R, 1/1), station 3 (3a, 2/2), station 4 (R, 3/3), station 5 (1/1), station 10 (R, 1/1), and station 11 (R, 2/3) with accompanying post-treatment reactions; no cancer metastasis was detected in two lymph nodes from station 7. Therefore, the patient achieved a pathological evaluation of MPR following neoadjuvant alectinib therapy. Moreover, we also performed NGS on the resected specimen to check for possible resistance mechanisms in the remaining active tumor. The NGS result showed MIR217HG-ALK fusion variant and no TP53 mutation, consistent with the finding of NGS analysis before alectinib treatment. Subsequent to surgery, adjuvant alectinib treatment was continued by the patient who has now maintained DFS for over 19 months without obvious adverse reactions noted (Figure 4).

## Discussion

A retrospective study conducted by Tatsuya Yoshida et al. unveiled that crizotinib exhibits varying sensitivity towards different ALK fusion variants (Yoshida et al., 2016), indicating the presence of heterogeneity in the response of ALK-TKIs to distinct ALK fusion partners. Thus, it holds immense significance to ascertain the specific types and breakpoints of ALK fusion variants for guiding clinical treatment decisions for patients. There are numerous techniques available for the detection of ALK fusion, and in comparison to traditional methods such as IHC, FISH, and RT-PCR, NGS detection enables more comprehensive detection of ALK fusion partners, facilitating the identification of a broader spectrum of novel ALK rearrangements, and is therefore a critical method for thorough ALK fusion analysis (Letovanec et al., 2018). RNA-based sequencing and whole transcriptome sequencing can serve as complementary tools to DNA-based NGS for the detection of rare ALK fusion variants (Benayed et al., 2019). Furthermore, the structural domain of ALK tyrosine kinase plays a pivotal role in driving tumorigenesis (Ou et al., 2020; Soda et al., 2007). In this case, a novel MIR217HG-ALK rearrangement was identified through NGS testing, which retained the intact kinase domain of ALK and potentially activated tumorigenesis, leading to sensitivity to ALK-TKIs. We have demonstrated that the patient harboring MIR217HG-ALK rearrangement exhibit a favorable response to neoadjuvant alectinib, achieving MPR, with a DFS exceeding 19 months.

Currently, alectinib has demonstrated superior clinical efficacy compared to crizotinib in advanced ALK-positive NSCLC (Camidge et al., 2019). However, the investigation of alectinib as neoadjuvant therapy for locally advanced ALK-positive NSCLC remains inadequate. A retrospective study revealed remarkable pathological response rate and safety profile of crizotinib in patients with ALK rearrangement undergoing neoadjuvant treatment (Zhang et al., 2019). Moreover, several clinical cases have reported the feasibility of utilizing alectinib as neoadjuvant therapy (Cao et al., 2023; Wang et al., 2023). The ongoing ALNEO clinical trial is also to evaluate the efficacy and safety of alectinib as neoadjuvant therapy in surgically resectable stage III ALK-positive NSCLC (Leonetti et al., 2021). These studies present promising prospects for the application of ALK-TKIs in neoadjuvant treatment.

However, neoadjuvant ALK-TKI therapy for patients with locally advanced unresectable N3-stage NSCLC harboring ALK rearrangement has only been reported in isolated cases (Zhai et al., 2023). Zhai X et al. documented a stage IIIB-N3 patient achieving qPCR after 5 months of neoadjuvant alectinib14, paralleling the treatment duration in our case. Additionally, Shi L et al. described two patients with stage IIB lung adenocarcinoma who achieved qPCR following long-course neoadjuvant alectinib (Shi et al., 2023). Given the advanced clinical stage (IIIB-N3) of the patient and the lack of established neoadjuvant treatment regimens or drug selection for novel ALK-rearranged NSCLC, the multidisciplinary team (MDT) at our institution opted for neoadjuvant alectinib, which has demonstrated superior efficacy over crizotinib in advanced ALK-rearranged disease (Camidge et al., 2019). After 3 months of alectinib treatment, imaging evaluation was performed. The surgeon recommended continued treatment to enable radical surgical resection. Consequently, the patient received alectinib for 5 months and met the surgical criteria. Surgery was performed successfully, and postoperative pathology revealed a MPR. However, residual cancer cells were still present in the primary lesion and lymph nodes of the resected specimen, and the patient had been initially diagnosed at a relatively advanced clinical stage. To potentially prolong the patient’s DFS and OS, the patient, after thorough discussion, opted for adjuvant alectinib treatment. Currently, DFS has exceeded 19 months. Unfortunately, financial constraints led the patient to forgo potential biomarker testing, such as circulating tumor DNA (ctDNA), opting solely for imaging evaluation, thereby limiting our study. In future clinical practice, a combined approach incorporating both imaging examinations and biomarker detection should be prioritized to enable earlier identification of disease recurrence or metastasis, thereby facilitating timely clinical interventions.

Our study, being a single-case report, necessitates further collection of clinical data from patients with the MIR217HG-ALK rearrangement to summarize clinical characteristics, treatment patterns, and conduct prognosis analyses. The current follow-up period for this case is 19 months, and we will continue to monitor the patient’s DFS and OS, along with treatment strategies post-recurrence or metastasis. Although we validated the findings with NGS on residual tissue, single-cell sequencing was not performed, limiting our exploration of the carcinogenic mechanisms of this novel fusion. Future efforts will focus on patients with MIR217HG-ALK rearrangement, aiming for a multi-center case study, dynamic monitoring of ctDNA in similar cases, and the development of a patient-derived xenograft (PDX) model to further investigate the oncogenic mechanisms of this ALK rearrangement.

## Conclusion

In conclusion, we have identified a novel MIR217HG-ALK rearrangement in NSCLC for the first time, thereby expanding the spectrum of ALK fusion variants. Moreover, we have provided preliminary evidence demonstrating the feasibility and safety of neoadjuvant alectinib in locally advanced unresectable NSCLC harboring MIR217HG-ALK rearrangement. We anticipate that prospective studies investigating the application of ALK-TKI in rare ALK-rearranged NSCLC, thereby offering more precise personalized treatment options for patients’ clinical management.

## Data Availability

The datasets for this article are not publicly available due to concerns regarding participant/patient anonymity. Requests to access the datasets should be directed to the corresponding author.
